# Characterization of a *Mapt* knock‐out rat: Sex‐dependent effects on LTP and object location memory

**DOI:** 10.1002/alz70855_102955

**Published:** 2025-12-23

**Authors:** Liam T Ralph, Ashish Kadia, Dan McElroy, Patrick Tidball, Kaylen M Young, Aiden E Glass, Meng Tian, John Georgiou, John G Howland, Graham L Collingridge

**Affiliations:** ^1^ Tanz Centre for Research in Neurodegenerative Diseases, Toronto, ON, Canada; ^2^ Lunenfeld‐Tanenbaum Research Institute, Toronto, ON, Canada; ^3^ University of Saskatchewan, Saskatoon, SK, Canada; ^4^ TANZ Centre for Research in Neurodegenerative Diseases, Toronto, ON, Canada; ^5^ University of Toronto, Toronto, ON, Canada

## Abstract

**Background:**

An emerging strategy for treating Alzheimer's disease is tau lowering therapeutics. These aim to reduce the levels of pathological tau species to prevent cognitive decline through approaches with RNA therapeutics, immunotherapy, small molecule inhibitors, or AAV‐based gene therapy. By studying the consequence of tau reduction in animal models one can understand the impact on learning and memory and physiological processes, potentially offering a way to optimize more targeted tau‐lowering therapeutics.

**Methods:**

Using our novel microtubule‐associated protein tau homozygous knock‐out (*Mapt* ^‐/‐^) rat model, we studied the impact of lack of tau on object‐location memory (OLM) in older adult (12‐to‐16‐month‐old) rats.

**Results:**

We have previously found that in the Schaffer collateral‐commissural pathway of older adult rats, deletion of tau enhances long‐term potentiation, the strengthening of synaptic efficacy, in female but not male *Mapt* ^‐/‐^ rats, relative to sex‐matched littermate WT rats (Ralph et al., 2023, CAN). In line with this enhanced synaptic plasticity, we now report that OLM is significantly improved in female *Mapt* ^‐/‐^ older adult rats relative to age‐matched female WT rats (*n* = 9 per group). This is while OLM is similar between WT and *Mapt* ^‐/‐^ male older adult rats (*n* = 7 per group). Furthermore, this effect is age‐dependent since neither female nor male *Mapt* ^‐/‐^ young adult rats (2‐to‐3‐month‐olds) exhibit any significant changes in OLM performance relative to sex‐matched WT rats (Young et al., 2025, CAN).

**Conclusion:**

Our findings provide evidence of a beneficial impact of long‐term tau deletion on synaptic plasticity and OLM, underscoring the potential for tailoring tau‐lowering therapeutics by age, sex and brain region once the underlying mechanism is fully elucidated.

